# HIF1A‐Associated Ferroptosis‐Related Gene Signatures Reveal Candidate Circulating Biomarkers in Diabetic Nephropathy

**DOI:** 10.1096/fba.2026-00173

**Published:** 2026-08-25

**Authors:** Yufeng Li, Jiao Bao, Rong Sun, Wenjie Mei, Yonghong Liu, Yan Bai, Chengxiang Hu, Hongying Jiang

**Affiliations:** ^1^ Department of Nephrology The Second People's Hospital of Qujing City Qujing Yunnan China; ^2^ The Second Affiliated Hospital of Kunming Medical University Kunming China

**Keywords:** diabetic nephropathies, ferroptosis, HIF1A, SLC2A14, SLC7A5

## Abstract

Diabetic nephropathy (DN) is a major microvascular complication of diabetes. Hypoxia‐inducible factor 1‐alpha (HIF1A) and ferroptosis contribute to the progression of DN, but the circulating biomarkers associated with these pathways are still unknown. In this study, the whole‐blood transcriptomic datasets in the Gene Expression Omnibus were combined with ferroptosis‐related genes from the FerrDb database and published literature. The differentially expressed genes of HIF1A and of DN were intersected, and then the feature selection with Boruta and SVM‐RFE was performed, followed by external validation, immune infiltration analysis, prediction of regulatory network, drug‐target prediction, and RT‐qPCR validation. We identified 773 HIF1A‐related genes, 1445 DN‐related genes, and 22 overlapping candidate genes. Using machine‐learning analysis, nine core genes were identified, including SLC7A5 and SLC2A14, which consistently upregulated in both training and validation datasets and corroborated by RT‐qPCR. Both genes were positively associated with activated natural killer cells and negatively associated with monocytes in estimated circulating immune‐cell composition analysis. Regulatory‐network and DrugBank analyses generated hypotheses regarding upstream miRNAs and potential compound interaction. These findings identify SLC7A5 and SLC2A14 as candidate circulating biomarkers related to HIF1A expression and ferroptosis‐related gene signatures in DN. Further large‐cohort and functional studies are required to confirm their diagnostic and mechanistic relevance.

## Graphical abstract

Workflow for identifying HIF1A‐expression‐associated ferroptosis‐related candidate circulating biomarkers in diabetic nephropathy using whole‐blood GEO transcriptomic datasets, ferroptosis‐related gene screening, exploratory machine‐learning prioritization, external validation, RT‐qPCR validation, and estimated circulating immune‐cell composition analysis.

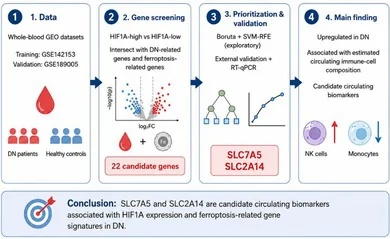

## 1 Introduction

Diabetic nephropathy (DN) or diabetic kidney disease is a common microvascular complication of diabetes and a cause of chronic kidney disease and end‐stage renal disease. While individuals with diabetes can achieve a slowing of kidney function decline with current therapies, such as glycemic control, blood pressure management, renin–angiotensin system blockade, sodium‐glucose cotransporter 2 inhibitors, and comprehensive cardiovascular risk reduction, many end up with further kidney injury [1, 2, 3]. Thus, it is important to identify molecular markers and mechanism targets to enable early diagnosis, risk stratification, and better therapeutic intervention.

Hypoxia and ferroptosis are gaining importance in the pathogenesis of DN. HIF1A encodes hypoxia‐inducible factor 1‐alpha, which is an oxygen‐regulated transcription factor that is associated with diabetic complications and diabetic kidney injury [4, 5, 6]. Ferroptosis is a form of regulated cell death characterized by lipid peroxidation and failure of antioxidant pathways that requires iron and is nonapoptotic. Experimental and review studies have shown that ferroptosis plays a role in renal tubular damage, podocyte damage, oxidative stress, inflammation, and fibrosis in DN [7, 8, 9, 10, 11]. Of note, the HIF‐1α/HO‐1 pathway has been shown to contribute to diabetic renal tubular injury, and it has been reported to crosstalk with ferroptosis [12].

The role of solute carrier genes in metabolic stress, nutrient transport and immune‐cell function is rapidly increasing [13]. SLC7A5 encodes LAT1, a large neutral amino acid transporter that is associated with amino acid uptake, metabolic reprogramming and activation of immune cells. The SLC2A14 belongs to the glucose transporter family, and may represent changes in glucose handling/metabolic adaptation. The role of SLC7A5 and SLC2A14 as circulating molecular signatures linked with DN, HIF1A expression and gene networks involved in ferroptosis have been poorly characterized.

The underlying genes involved in DN that are associated with HIF1A and how they interact with circulating immune‐cell proportion, however, are not fully defined. So, the main question of the present study was: Which HIF1A‐associated ferroptosis‐related genes are altered in DN and can serve as potential circulating biomarkers associated with immune dysregulation? To answer this question, we combined GEO transcriptomic data with ferroptosis‐associated genes from FerrDb [14] and performed machine learning‐based feature selection, external validation, estimated circulating immune‐cell composition analysis, prediction of regulatory networks, prediction of drug‐targets, and validation by RT‐qPCR. The goal was to identify markers of ferroptosis associated with HIF1A in DN as candidates and to give clues for future mechanistic studies in a translational context.

## 2 Materials and Methods

### 2.1 Gene‐Expression Data Collection

Gene‐expression profiles were obtained from the Gene Expression Omnibus (GEO; https://www.ncbi.nlm.nih.gov/geo/). The training dataset GSE142153 (platform GPL6480) included whole‐blood transcriptomic data from 23 patients with DN and 10 healthy controls. The external validation dataset GSE189005 also comprised whole‐blood transcriptomic data and included 8 patients with DN and 9 healthy controls. A total of 564 ferroptosis‐related genes (FRGs) were obtained from FerrDb (http://www.zhounan.org/ferrdb/) and published annotations/literature, including Table S1 and the reference indexed by PMID: 37603208. The ferroptosis association in this study was inferred from FerrDb and published ferroptosis‐related gene annotations. Ferroptosis activity was not directly measured in the GEO datasets or RT‐qPCR samples. In the public GEO datasets, DN and control groups were defined according to the original dataset annotations. DN samples were annotated as diabetic nephropathy patient samples, whereas control samples were annotated as healthy or non‐DN control blood samples. For the RT‐qPCR cohort, DN patients were diagnosed according to clinical history of diabetes with evidence of nephropathy based on the treating institution's diagnostic criteria. Controls were individuals without DN.

### 2.2 Differential Expression Analysis of HIF1A‐Related Genes

The Wilcoxon rank‐sum test was used to compare HIF1A expression between DN patients and healthy controls in the training dataset, with *p* < 0.05 considered statistically significant. HIF1A expression was visualized using the R package ggpubr (v0.6.0; https://CRAN.R‐project.org/package=ggpubr). To identify HIF1A‐related differentially expressed genes (HDEGs), DN samples in the training dataset were stratified into HIF1A high‐ and low‐expression groups using the median HIF1A expression value as the cutoff. Differential expression analysis between these two groups was performed using the R package limma (v3.58.1; PMID: 25605792). Genes with|log2 fold change (log2FC)|> 0.5 and *p* < 0.05 were considered HDEGs. Volcano plots were generated using ggplot2 (v3.5.1; PMID: 35751589). The top 10 upregulated and top 10 downregulated genes ranked by log2FC were displayed in heatmaps generated using ComplexHeatmap (v2.18.0; PMID: 38868715).

### 2.3 Differential Expression Analysis Between DN Patients and Controls and Identification of Candidate Genes

Differentially expressed genes (DEGs) between DN patients and healthy controls in the training dataset were identified using limma (v3.58.1), with |log2FC| > 0.5 and *p* < 0.05 as selection criteria. Volcano plots were generated using ggplot2, and heatmaps of the top 10 upregulated and top 10 downregulated genes were produced using ComplexHeatmap. To identify HIF1A‐associated ferroptosis‐related genes in DN, an intersection analysis was performed among DEGs, HDEGs, and FRGs. The overlap was visualized using ggvenn (v0.1.10; PMID: 35845045), and the intersecting genes were defined as candidate genes for downstream analyses [15].

### 2.4 Functional Enrichment and Protein–Protein Interaction Analysis

Gene Ontology (GO) and Kyoto Encyclopedia of Genes and Genomes (KEGG) enrichment analyses were performed using clusterProfiler (v4.10.1; PMID: 34557778) to explore the biological functions and pathways associated with the candidate genes. Terms or pathways with *p* < 0.05 were considered significantly enriched. Enrichment results were visualized using enrichplot (v1.20.3; PMID: 36618352). GO analysis included biological process (BP), cellular component (CC), and molecular function (MF) categories. Protein–protein interaction (PPI) analysis was performed using the STRING database (http://www.string‐db.org/) with a minimum confidence score of 0.4. The resulting network was imported into Cytoscape (v3.9.1; PMID: 33149752) for visualization, and genes without predicted interactions were excluded from the final network.

### 2.5 Machine‐Learning Feature Selection

Machine‐learning analyses were used as exploratory candidate‐gene prioritization methods rather than definitive diagnostic model development. Boruta feature selection was performed using the R package Boruta (v8.0.0), with *p* = 0.05 and maxRuns = 500. Genes classified as “Confirmed” were retained. SVM‐RFE was performed using the R package e1071 (v1.7.14). Model accuracy was recorded across feature subsets, and the feature combination with the highest accuracy was selected. To reduce overinterpretation and potential overfitting, only genes overlapping between Boruta and SVM‐RFE were retained as core genes, and their expression was further evaluated in an external validation dataset and by RT‐qPCR. Because of the limited sample size, these analyses were considered exploratory.

### 2.6 Expression Validation of Core Genes

The Wilcoxon rank‐sum test was used to compare the expression levels of core genes between DN patients and healthy controls in both the training and external validation datasets. Boxplots were generated using ggplot2. Core genes with significant differential expression (*p* < 0.05) and consistent expression trends in both datasets were defined as key genes.

### 2.7 Immune‐Cell Composition Analysis

Immune infiltration of 22 immune cell types was estimated in the training dataset using the CIBERSORT algorithm (PMID: 29344893), based on the corresponding R implementation. The relative abundance of each immune cell type in each sample was visualized using ggplot2. Differences in immune cell infiltration between DN patients and healthy controls were evaluated using the Wilcoxon rank‐sum test. Only immune cell types detected in at least 50% of samples were included in the comparative analysis, and cell types with zero infiltration values across samples were excluded. Spearman correlation analysis was performed to assess associations between key genes and differentially estimated circulating immune‐cell subsets using the R package psych (v2.4.3; https://CRAN.R‐project.org/package=psych). Correlations with|correlation coefficient|> 0.3 and *p* < 0.05 were considered significant and were visualized using ggplot2.

### 2.8 Molecular Regulatory Network and Drug‐Target Prediction

To explore potential upstream regulatory mechanisms of the key genes, miRNA‐mRNA regulatory relationships were predicted using multiMiR (v1.24.0; PMID: 25063298) by integrating results from DIANA‐Tools, miRDB, and EIMMo/ELMMo databases. Predicted miRNA‐mRNA networks were visualized using Cytoscape. Transcription factors (TFs) potentially regulating the key genes were identified using the hTFtarget database (https://guolab.wchscu.cn/hTFtarget/#!/). The TF‐mRNA network was constructed and visualized using Cytoscape. Potential drugs interacting with proteins encoded by the key genes were predicted using the DrugBank database (https://go.drugbank.com/). Drug‐gene interaction networks were constructed using Cytoscape to identify candidate compounds for further experimental evaluation.

### 2.9 RT‐qPCR Experimental Validation

RT‐qPCR was performed to validate the expression of candidate circulating biomarkers. Fifty fresh blood samples were collected at The Second People's Hospital of Qujing City, Yunnan Province, including 25 control samples (Nos. 1–25) and 25 DN samples (Nos. 26–50). Total RNA was extracted using TRIzol reagent (Vazyme, China) according to the manufacturer's instructions [16]. Reverse transcription was performed using the HP All‐in‐one qRT Master Mix II kit (YunGen, China). The qPCR reaction volume was 20 μL and contained 10 μL of 2× Universal Blue SYBR Green qPCR Master Mix, 1 μL each of forward and reverse primers (10 micromolar), 2 μL of cDNA template, and 6 μL of RNase‐free water. Cycling conditions were as follows: initial denaturation at 95°C for 30 s, followed by 39 cycles of 95°C for 5 s and 60°C for 30 s. Amplification and melting curves were recorded. Reactions were performed on a CFX Connect Real‐Time PCR System (Bio‐Rad, USA), and relative expression was calculated using the 2^−ΔΔCT^ method. Primer sequences are listed in Table S2.

### 2.10 Statistical Analysis

All bioinformatic analyses were performed using R software (v4.2.2). Between‐group comparisons were conducted using the Wilcoxon rank‐sum test or Student's *t*‐test, as appropriate. Spearman correlation analysis was used to examine relationships between key genes and immune cell proportions. Unless otherwise specified, raw *p*‐values were used for exploratory differential‐expression, enrichment, and correlation analyses, with *p* < 0.05 considered statistically significant. Where multiple‐testing correction was available in the relevant R package output, adjusted *p*‐values/FDR values were also recorded and provided in Supporting Information (Tables [Link], [Link], [Link], [Link], [Link], [Link]). Because this study was exploratory and based on relatively small datasets, findings were interpreted cautiously and prioritized for external and RT‐qPCR validation.

## 3 Results

### 3.1 Identification of HIF1A‐Associated Ferroptosis‐Related Candidate Genes

**FIGURE 1:**
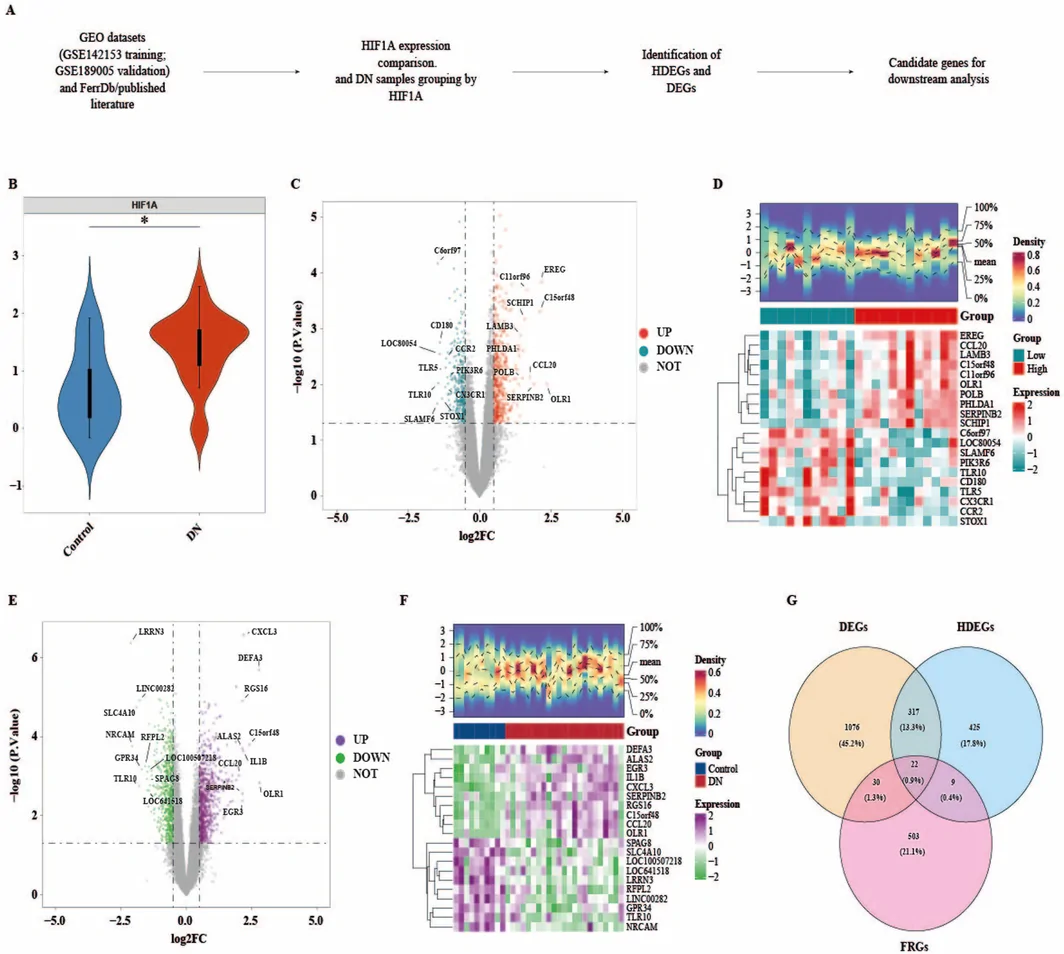
Identification of HIF1A‐associated ferroptosis‐related candidate genes in DN. (A) HIF1A expression in DN and control samples. (B) Volcano plot of HDEGs. (C) Heatmap of top HDEGs. (D) Volcano plot of DEGs between DN and controls. (E) Heatmap of top DEGs. (F) Venn diagram showing the overlap among DEGs, HDEGs, and FRGs. DEG, differentially expressed gene; DN, diabetic nephropathy; FRG, ferroptosis‐related gene; HDEG, HIF1A‐related differentially expressed gene.

*HIF1A* expression was significantly higher in DN patients than in healthy controls in the training dataset (*p* < 0.05; Figure 1A). Using the median *HIF1A* expression value (1.60) as the cutoff, the 23 DN samples were divided into *HIF1A* high‐ and low‐expression groups. Differential expression analysis identified 773 HDEGs, including 425 upregulated and 348 downregulated genes in the *HIF1A* high‐expression group (*p* < 0.05 and |log2FC| > 0.5; Figure 1B and Table S3). A heatmap of the top 10 upregulated and top 10 downregulated genes showed distinct expression patterns between the two groups (Figure 1C). These results indicate that *HIF1A* expression is associated with broad transcriptional alterations in DN.

Differential expression analysis between DN patients and healthy controls identified 1445 DEGs in the training dataset, including 707 upregulated and 738 downregulated genes in DN (Figure 1D and Table S4). The heatmap illustrated distinct expression patterns of the top DEGs between DN and control samples (Figure 1E). Intersection analysis among DEGs, HDEGs, and FRGs identified 22 candidate *HIF1A*‐associated ferroptosis‐related genes (Figure 1F). These genes were selected for subsequent functional, network, and biomarker analyses.

### 3.2 Functional Enrichment and PPI Analysis of Candidate Genes

**FIGURE 2:**
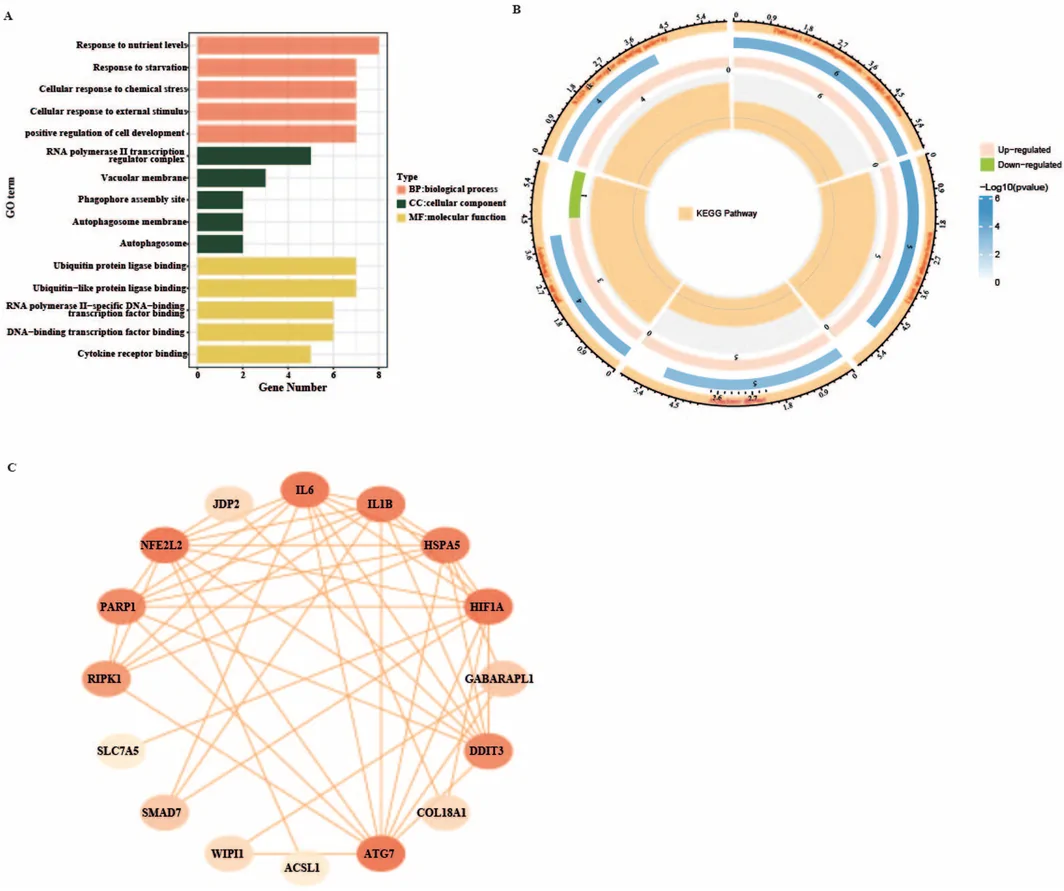
Functional enrichment and PPI analysis of candidate genes. (A) GO enrichment analysis. (B) KEGG enrichment analysis. (C) PPI network of candidate genes. GO, Gene Ontology; KEGG, Kyoto Encyclopedia of Genes and Genomes; PPI, protein–protein interaction.

GO enrichment analysis of the 22 candidate genes identified 1128 significantly enriched terms (*p* < 0.05; Table S5), including 1023 BP terms, 20 CC terms, and 85 MF terms. The top BP terms included response to nutrient levels, response to starvation, cellular response to chemical stress, cellular response to external stimulus, and positive regulation of cell development. These terms suggest that the candidate genes may participate in metabolic stress responses and adaptive cellular programs in DN. The enriched CC terms were mainly related to intracellular and transcription‐associated components, whereas MF terms included ubiquitin protein ligase binding, ubiquitin‐like protein ligase binding, RNA polymerase II‐specific DNA‐binding transcription factor binding, DNA‐binding transcription factor binding, and cytokine receptor binding (Figure 2A). KEGG enrichment analysis identified 57 significantly enriched pathways (*p* < 0.05; Table S6). The top enriched pathways included pathways of neurodegeneration—multiple diseases, lipid and atherosclerosis, Alzheimer disease, autophagy—animal, and the NOD‐like receptor signaling pathway (Figure 2B). Overall, enrichment analysis indicated that the candidate genes were mainly involved in cellular stress responses, transcriptional regulation, autophagy, lipid‐associated pathways, and inflammatory signaling, supporting their potential relevance to DN pathogenesis.

The PPI network contained 16 interconnected proteins and 47 interaction pairs (Figure 2C). *HIF1A* interacted with several proteins, including *DDIT3*, *HSPA5*, and *IL6*, suggesting that *HIF1A* may occupy a central position within the candidate‐gene network.

### 3.3 Identification and Validation of Key Genes by Machine Learning

**FIGURE 3:**
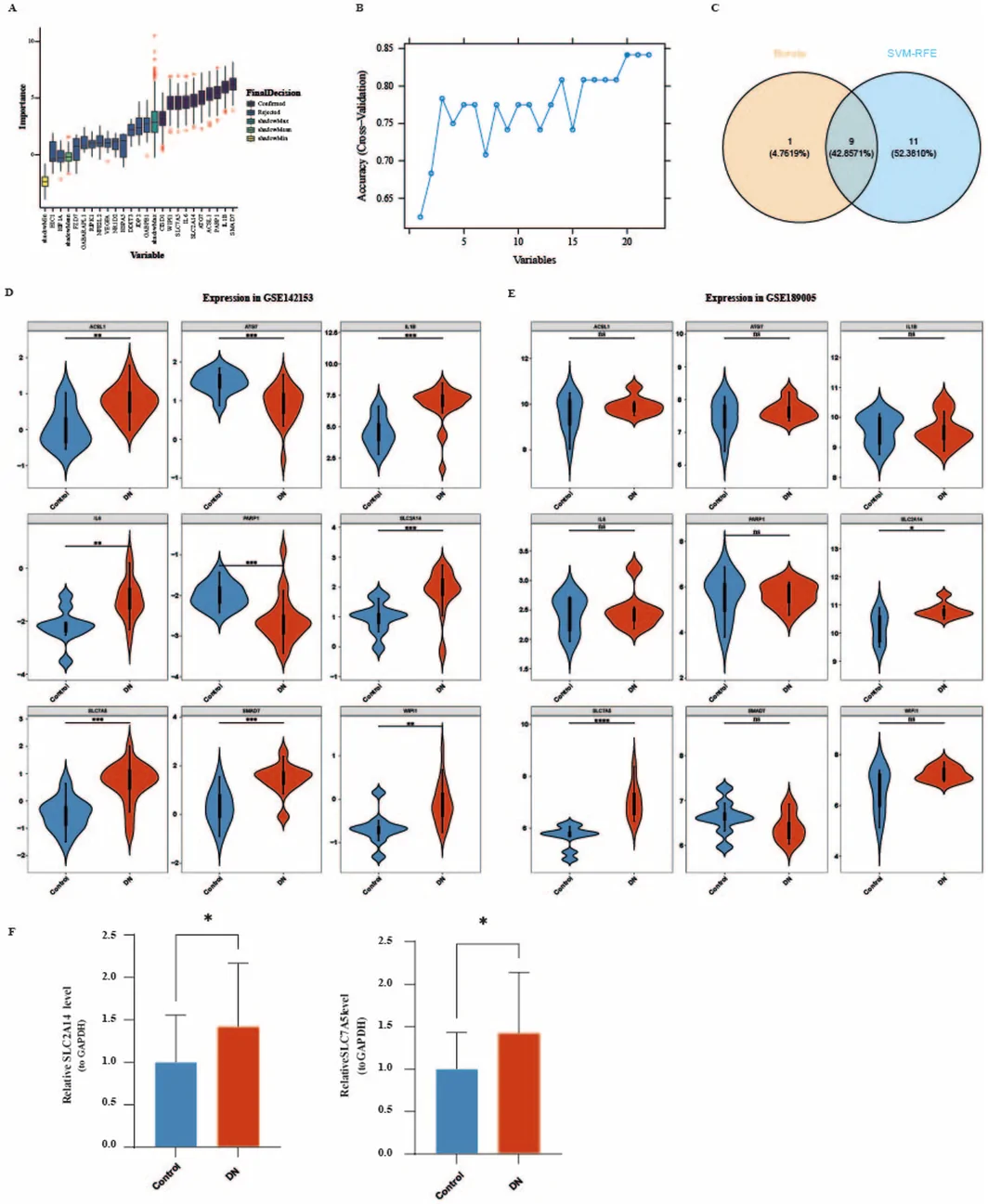
Machine‐learning identification and validation of key genes. (A) Boruta feature‐selection results. (B) SVM‐RFE results. (C) Overlap between Boruta and SVM‐RFE feature genes. (D) Expression validation in GSE142153. (E) Expression validation in GSE189005. (F) RT‐qPCR validation of SLC7A5 and SLC2A14. DN, diabetic nephropathy; SVM‐RFE, support vector machine recursive feature elimination.

To prioritize the candidate genes, two machine‐learning algorithms were applied. Boruta analysis identified 10 confirmed feature genes: *SMAD7*, *SLC7A5*, *IL1B*, *ACSL1*, *IL6*, *ATG7*, *WIPI1*, *SLC2A14*, *PARP1*, and *CISD1* (Figure 3A).

SVM‐RFE analysis selected 20 feature genes at the highest model accuracy: *IL1B*, *SMAD7*, *SLC7A5*, *ATG7*, *PARP1*, *SLC2A14*, *GABPB1*, *WIPI1*, *ACSL1*, *IL6*, *GABARAPL1*, *DDIT3*, *VEGFA*, *NR1D2*, *NFE2L2*, *RIPK1*, *HSPA5*, *JDP2*, *HIF1A*, and *FZD7* (Figure 3B). The intersection of Boruta and SVM‐RFE feature genes yielded nine core genes: *SMAD7*, *SLC7A5*, *IL1B*, *ACSL1*, *IL6*, *ATG7*, *WIPI1*, *SLC2A14*, and *PARP1* (Figure 3C). Expression validation in both the training and external validation datasets showed that *SLC7A5* and *SLC2A14* were significantly upregulated in DN patients compared with controls (*p* < 0.05; Figure 3D,E). These two genes were therefore defined as key genes for subsequent analyses. RT‐qPCR analysis confirmed that *SLC7A5* and *SLC2A14* expression levels were significantly higher in the DN group than in the control group (*p* < 0.05; Figure 3F). This expression pattern was consistent with the results obtained from the training and validation transcriptomic datasets.

### 3.4 Immune‐Cell and Regulatory Analysis of SLC7A5 and SLC2A14

**FIGURE 4:**
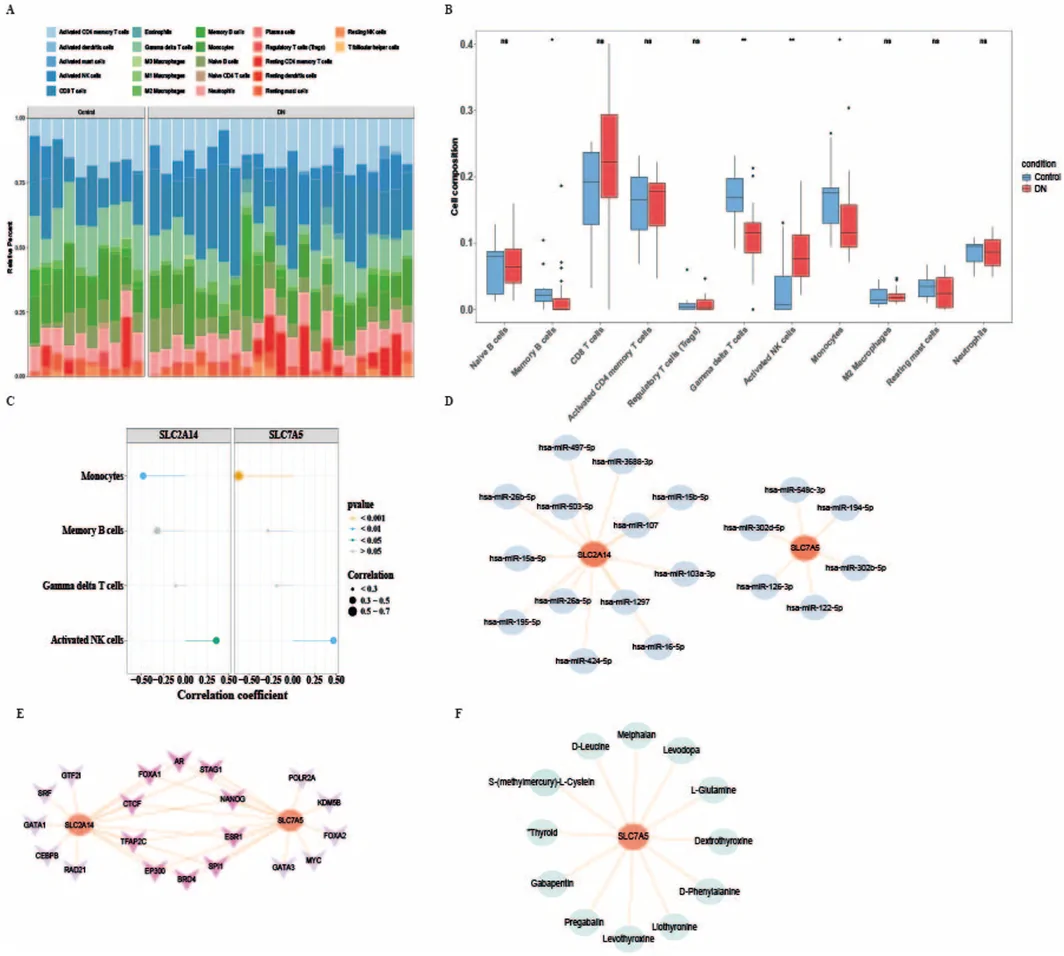
Estimated circulating immune‐cell composition and regulatory analysis of SLC7A5 and SLC2A14. (A) Estimated immune‐cell proportions. (B) Differential immune‐cell proportions between DN and controls. (C) Correlations between key genes and differential immune cells. (D) miRNA‐mRNA network. (E) TF‐mRNA network. (F) Drug‐gene interaction network. DN, diabetic nephropathy; TF, transcription factor.

CIBERSORT‐based estimated circulating immune‐cell composition analysis estimated the relative proportions of 22 immune cell types in DN and control samples (Figure 4A).

Comparative analysis identified four immune cell types that differed significantly between DN patients and healthy controls (*p* < 0.05): memory B cells, gamma‐delta T cells, activated natural killer (NK) cells, and monocytes (Figure 4B). DN samples showed a higher proportion of activated NK cells and lower proportions of monocytes, gamma‐delta T cells, and memory B cells, indicating immune imbalance in DN.

Correlation analysis showed that *SLC7A5* and *SLC2A14* were positively correlated with activated NK cells (correlation coefficient > 0.3, *p* < 0.05) and negatively correlated with monocytes (correlation coefficient < −0.4, *p* < 0.01; Figure 4C). These results suggest that *SLC7A5* and *SLC2A14* may be associated with immune‐cell remodeling in DN. To investigate potential regulatory mechanisms, miRNA‐mRNA and TF‐mRNA networks were constructed for *SLC7A5* and *SLC2A14*. The miRNA‐mRNA network predicted 19 miRNAs targeting *SLC7A5* and/or *SLC2A14* (Figure 4D). For example, hsa‐miR‐103a‐3p was predicted to target *SLC2A14*, whereas hsa‐miR‐122‐5p was predicted to target *SLC7A5*. The TF‐mRNA network identified 20 predicted transcription factors associated with the key genes (Figure 4E). Both *SLC2A14* and *SLC7A5* were predicted to be regulated by transcription factors such as *CTCF*, *TFAP2C*, and *NANOG*. Additional gene‐specific TFs included *GTF2I*, *GATA1*, and *SRF* for *SLC2A14*, and *KDM5B*, *MYC*, and *POLR2A* for *SLC7A5*. DrugBank‐based prediction identified 11 potential drugs interacting with *SLC7A5*, whereas no DrugBank‐supported compound was predicted for *SLC2A14* in this analysis (Figure 4F). Among the predicted compounds, levodopa was identified as a potential *SLC7A5*‐interacting drug. These predictions require experimental validation before therapeutic relevance can be inferred.

## 4 Discussion

This study combined bioinformatics analysis, machine‐learning feature selection, external validation, immune‐cell composition analysis, regulatory‐network prediction, drug‐target prediction, and RT‐qPCR validation to identify *HIF1A*‐associated ferroptosis‐related genes in diabetic nephropathy (DN). Among the screened candidates, *SLC7A5* and *SLC2A14* were consistently upregulated in DN in both public whole‐blood transcriptomic datasets and clinical RT‐qPCR validation. These findings indicate that both genes may be potential circulating biomarkers linked to hypoxic, metabolic, ferroptotic, and immune changes in DN.

SLC7A5 and SLC2A14 are known to be HIF1A‐expression‐associated, but the two are not known to have been used together as circulating candidate circulating biomarkers for ferroptosis in DN to date, as far as we know. SLC7A5 has been associated with amino acid transport, metabolic reprogramming, and activation of immune cells; SLC2A14 might be related to glucose transporter‐related metabolic adaptation. They are consistently elevated in whole‐blood datasets and were validated using RT‐qPCR, indicating that they are likely markers of metabolic and immune dysfunctions which occur systemically during DN, and not only in the kidney.

Despite improved glycemic control, blood‐pressure management, renin–angiotensin system blockade, and sodium‐glucose cotransporter 2 inhibitor therapy, diabetic kidney disease and end‐stage kidney disease remain significant chronic complications of diabetes, with an increasing clinical burden [1, 2, 3]. The pathogenesis of DN includes metabolic stress, oxidative stress, inflammation, hypoxia, and regulated cell death [4, 5, 6]. Renal hypoxia has been implicated in DN progression, and *HIF1A* is one of the key transcriptional regulators of cellular adaptation to hypoxia [4, 5, 6]. Diabetic kidney injury has also been linked to ferroptosis, a regulated nonapoptotic form of cell death that is iron‐dependent and characterized by lipid peroxidation and antioxidant depletion [7, 8, 9, 10]. Previous studies reported that ferroptosis may exacerbate diabetic renal tubular injury via the HIF‐1α/HO‐1 pathway, and that disruption of iron metabolism, including *ZIP14*‐associated iron deposition, may contribute to ferroptotic injury in DN models [11, 12]. These findings support the relevance of *HIF1A*‐associated ferroptosis‐related genes as targets for further investigation in DN.

In this study, 22 candidate genes were identified at the intersection of DN‐related DEGs, *HIF1A*‐associated DEGs, and ferroptosis‐related genes [14, 17, 18, 19]. Functional enrichment suggested involvement in nutrient response, starvation response, cellular chemical‐stress response, autophagy, lipid‐related pathways, and inflammatory signaling, which is consistent with the metabolic and oxidative‐stress environment of DN. Exploratory machine‐learning‐based candidate‐gene prioritization identified *SLC7A5* and *SLC2A14* as consistently upregulated genes. *SLC7A5* encodes a large neutral amino acid transporter involved in amino acid transport and cellular metabolic regulation [20, 21, 22, 23], whereas *SLC2A14* belongs to the solute carrier 2 family and may be related to glucose‐handling or metabolic changes [24]. Immune‐cell composition analysis showed that both genes were positively correlated with activated NK cells and negatively correlated with monocytes, suggesting a possible relationship with systemic immune‐cell changes in DN [25, 26, 27, 28]. Because these results were obtained from whole‐blood transcriptomic data, they should be interpreted as circulating immune‐cell signatures rather than direct evidence of renal circulating immune‐cell composition. DrugBank prediction of the interaction between levodopa and *SLC7A5* was hypothesis‐generating only and requires experimental confirmation before therapeutic relevance can be inferred [26].

In conclusion, this study identified SLC7A5 and SLC2A14 as candidate circulating biomarkers associated with HIF1A expression and ferroptosis‐related gene signatures in DN. Their upregulation in whole‐blood transcriptomic datasets and RT‐qPCR validation, together with their associations with estimated circulating immune‐cell composition, suggest potential relevance to systemic metabolic and immune alterations in DN. However, these findings remain exploratory and require validation in larger independent cohorts, renal tissue datasets, and functional experiments.

## Author Contributions

Y.L. conceived and designed the study. J.B. and R.S. performed the experiments and exploratory analyses. W.M., Y.L., Y.B., and C.H. collected and analyzed the data. J.B. and R.S. drafted the manuscript. Y.L. supervised the study and critically revised the manuscript. H.J. contributed to data interpretation and manuscript revision. All authors reviewed and approved the final manuscript.

## Funding

This work was supported by the Scientific Research Fund Project of the Yunnan Provincial Department of Education (2024 J1631) and the Internal Research Project of the Second People's Hospital of Qujing City (Grant No. 2023ynkt07).

## Ethics Statement

The study protocol was approved by the Ethics Committee of The Second People's Hospital of Qujing City, Yunnan Province (Approval No. Rental Review 2023‐031 [Science]‐01).

## Consent

Informed written consent was obtained from the subjects before blood collection.

## Conflicts of Interest

The authors declare no conflicts of interest.

## Supporting information


**Table S1:** List of the 564 ferroptosis‐related genes obtained from FerrDb and published ferroptosis‐related gene annotations.

**Table S2:** Primer sequences used for RT‐qPCR validation of the candidate circulating biomarkers.

**Table S3:** HIF1A‐related differentially expressed genes identified between the HIF1A high‐ and low‐expression groups of patients with diabetic nephropathy in the GSE142153 training dataset.

**Table S4:** Differentially expressed genes identified between patients with diabetic nephropathy and healthy controls in the GSE142153 training dataset.

**Table S5:** Gene Ontology enrichment analysis of the 22 candidate HIF1A‐associated ferroptosis‐related genes. BP, biological process; CC, cellular component; GO, Gene Ontology; MF, molecular function.

**Table S6:** Kyoto Encyclopedia of Genes and Genomes pathway enrichment analysis of the 22 candidate HIF1A‐associated ferroptosis‐related genes. KEGG, Kyoto Encyclopedia of Genes and Genomes.

## Data Availability

All the data are available in the manuscript and can be acquired from the corresponding author upon special request.
